# Blog and Podcast Watch: Orthopedic Emergencies

**DOI:** 10.5811/westjem.2017.1.33197

**Published:** 2017-03-14

**Authors:** Andrew Grock, Salim Rezaie, Anand Swaminathan, Alice Min, Kaushal H. Shah, Michelle Lin

**Affiliations:** * Olive View, UCLA Medical Center, Department of Emergency Medicine, Sylmar, California; † Keck School of Medicine of the University of Southern California and the Los Angeles County + USC Medical Center, Department of Emergency Medicine, Los Angeles, California; ‡ Greater San Antonio Emergency Physicians, Department of Emergency Medicine, San Antonio, Texas; § NYU/Bellevue Hospital, Ronald O. Perelman Department of Emergency Medicine, New York, New York; ¶ University of Arizona, College of Medicine, Department of Emergency Medicine, Tucson, Arizona; || Icahn School of Medicine, Mount Sinai Hospital, Department of Emergency Medicine, New York, New York; # University of California San Francisco, Department of Emergency Medicine, San Francisco, California

## Abstract

**Introduction:**

The *WestJEM* Blog and Podcast Watch presents high quality open-access educational blogs and podcasts in emergency medicine (EM) based on the ongoing ALiEM Approved Instructional Resources (AIR) and AIR-Professional series. Both series critically appraise resources using an objective scoring rubric. This installment of the Blog and Podcast Watch highlights the topic of orthopedic emergencies from the AIR series.

**Methods:**

The AIR series is a continuously building curriculum that follows the Council of Emergency Medicine Residency Directors (CORD) annual testing schedule. For each module, relevant content is collected from the top 50 Social Media Index sites published within the previous 12 months, and scored by eight AIR board members using five equally weighted measurement outcomes: Best Evidence in Emergency Medicine (BEEM) score, accuracy, educational utility, evidence based, and references. Resources scoring ≥30 out of 35 available points receive an AIR label. Resources scoring 27–29 receive an honorable mention label, if the executive board agrees that the post is accurate and educationally valuable.

**Results:**

A total of 87 blog posts and podcasts were evaluated. Key educational pearls from the three AIR posts and the 14 honorable mentions are summarized.

**Conclusion:**

The *WestJEM* Blog and Podcast Watch series is based on the AIR and AIR-Pro series, which attempts to identify high quality educational content on open-access blogs and podcasts. This series provides an expert-based, post-publication curation of educational social media content for EM clinicians with this installment focusing on orthopedic emergencies.

## BACKGROUND

Despite the rapid rise of social media educational content available through blogs and podcasts in emergency medicine (EM),^1^ identification of quality resources for educators and learners has only received preliminary progress.^2^–^4^ In 2008 the Accreditation Council for Graduate Medical Education endorsed a decrease in synchronous conference experiences for EM residency programs by up to 20% in exchange for asynchronous learning termed individualized interactive instruction.^5^

To address this need, the Academic Life in Emergency Medicine (ALiEM) Approved Instructional Resources (AIR) Series and AIR-Pro Series were created in 2014 and 2015, respectively, to help EM residency programs identify quality online content specifically on social media.^6^,^7^ Using an expert-based, crowd-sourced approach, these two programs identify trustworthy, high quality, educational blog and podcast content. For the *WestJEM* Blog and Podcast Watch, summaries of these posts are written by the AIR and AIR-Pro Series’ editorial boards.^8^,^9^

This installment from the AIR Series summarizes the highest scoring social media educational resources on orthopedic emergencies.

## METHODS

### Topic Identification

The AIR series is a continuously building curriculum based on the CORD testing schedule (http://www.cordtests.org/).

### Inclusion and Exclusion Criteria

A search of the 50 most frequently visited sites per the Social Media Index^10^ was conducted for resources relevant to orthopedic emergencies, published within the previous 12 months. The search, conducted from March – May 2015, included blog posts and podcasts written in English for scoring by our expert panel.

### Scoring

Extracted posts were scored without blinding by eight reviewers from the AIR Editorial Board, which is comprised of EM core faculty from various U.S. medical institutions. The scoring instrument contains five measurement outcomes using seven-point Likert scales: Best Evidence in Emergency Medicine (BEEM) score, accuracy, educational utility, evidence based, and references (Table).^11^ More detailed methods are described in the original description of the AIR series.^6^,^7^ Board members with any role in the production of a reviewed resource recused him/herself from grading that resource.

### Data Analysis

Resources with a mean evaluator score of ≥ 30 points (out of a maximum of 35) are awarded the AIR label. Resources with a mean score of 27–29 and deemed accurate and educationally valuable by the reviewers are given the honorable mention label.

## RESULTS

We initially included a total of 87 blog posts and podcasts. Key educational pearls from the three AIR posts, and the 14 honorable mentions are described.

### AIR Content

#### 1. Bryant R. More Dogma: Epinephrine in Digital Nerve Blocks. *REBEL EM*. (September 3, 2015). http://rebelem.com/more-dogma-epinephrine-in-digital-nerve-blocks/

This post summarizes a 2015 review article that presents the evidence against the medical myth that epinephrine use in digits is dangerous.^12^

Take Home Points: The evidence for the medical myth involved 50 cases of digit necrosis after local anesthesia prior to 1949. After 1949, doctors no longer mixed their own epinephrine and lidocaine solutions, and no further reports of necrosis exist. In these original 50 cases, only 21 used epinephrine and only four had a known epinephrine concentration. In 23 studies since, 2,797 digital nerve blocks with epinephrine have been reported with no complications attributable to the use of epinephrine. Furthermore, of the 186 patients with accidental epinephrine injection from auto-injectors, an epinephrine dose over 100 times stronger than the dose used in local anesthetics, there were zero cases of digit necrosis. Though some patients received reversal agents, only four patients had documented ischemia, two of which resolved within two hours. For patients with poor circulation, the data is less robust. Nonetheless, the blog authors conclude that epinephrine is probably safe in high-risk patients such as those with uncontrolled hypertension, pheochromocytoma, hyperthyroidism, poor digital circulation, peripheral vascular disease, and diabetes. Phentolamine has been shown to decrease the duration of vasoconstriction.

#### 2. Grayson A. A St. Emlyn’s Fascia Iliaca Block Update. *St Emlyn’s*. (January 22, 2016). http://stemlynsblog.org/fib-virgil/

This post reviews the utility of and instructions for fascia iliaca block for hip fractures.

Take Home Points: Hip fractures tend to occur in the elderly population, a group with increased potential side effects from opioids. Several papers have demonstrated the safety and efficacy of one alternative to opioids, the fascia iliaca block. The femoral nerve has two fascial sheaths and is located lateral to the artery. A landmark technique or an ultrasound-guided approach can be used, as outlined in the associated videos. Notably 30–40 mL of diluted anesthetic should be administered in this field block. Given the ease and safety of this technique, this presents an excellent alternative to opioids for pain control of hip fractures especially in elderly patients.

#### 3. Ritcey B. Bond C. SGEM#138: Hip to Be Blocked – Regional Nerve Blocks for Hip and Femoral Neck Fractures. *Skeptics Guide to EM*. (November 29, 2015). http://thesgem.com/2015/11/sgem138-hip-to-be-blocked-regional-nerve-blocks-for-hip-and-femoral-neck-fractures/

This blog post critiques a 2015 *Canadian Journal of Emergency Medicine* systematic review of regional nerve blocks for hip and femoral neck fractures.^13^

Take Home Points: Although the featured systematic review publication of nine studies had small sample sizes, moderate-to-high bias, and heterogeneous methodologies, the blog’s authors concur with the publication’s conclusion that nerve blocks seem to be an effective alternative or adjunct to standard pain therapy for hip or femoral neck fractures. These studies included three different approaches to a nerve block: traditional femoral nerve block, 3-in-1 femoral nerve block, and fascia iliaca compartment block. It is therefore reasonable to offer one of these regional nerve blocks to patients with an isolated hip fracture for pain control.

### Honorable Mention

#### 1. Heimann M and Barolotta K. Septic Joint: Reminders, Updates and Pitfalls. *EM Docs*. (May 9, 2015). http://www.emdocs.net/septic-joint-reminders-updates-and-pitfalls/

This blog post reviews key elements and pitfalls in the diagnosis and management of septic arthritis.

Take Home Points: Evaluation of a painful joint poses multiple diagnostic challenges. Cases of septic arthritis are typically monoarticular, although they can be polyarticular in up to 20% of cases. A fever is present in only 50% of cases. The presence of generalized tenderness with painful limitation of both active and passive range of motion indicates true joint involvement. Plain radiography, ultrasound, computed tomography (CT), magnetic resonance imaging (MRI), and radionuclide scanning lack specificity and are unable to differential septic arthritis from other inflammatory etiologies. Serum markers have limited sensitivity and specificity and cannot exclusively rule out septic arthritis.

Synovial fluid analysis should include inspection for color, clarity, and viscosity, as well as testing for white blood cell count (WBC), crystal analysis, Gram stain, and bacterial culture. Interpretation of this joint fluid may be problematic as a low WBC count may be seen in early presentations of septic arthritis, especially when caused by methicillin-resistant *S. aureus*, and elevated WBC count (>50,000/mm^3^) may be seen in non-infectious causes of arthritis. A WBC count >100,000/mm, however, carries a likelihood ratio (LR) of 28 for a septic joint. Early identification and prompt treatment including empiric antibiotic coverage and drainage of the infected joint is critical to minimize mortality and morbidity.

#### 2. Moleno R, Venezia, M. Open Fractures. *EM Docs*. (December 31, 2015). http://www.emdocs.net/open-fractures-pearls-and-pitfalls/

This post discusses open fracture classification and the evidence supporting traditional and current therapies.

Take Home Points: The Gustilo-Anderson classification system can guide patient management and is based on the size of the skin defect and the degree of soft tissue injury and contamination. The Mangled Extremity Severity Score (MESS) estimates viability of the extremity in order to predict empiric amputation versus attempted salvage of the extremity.

After an evaluation of the airway, breathing, and circulation with initiation of blood products or coagulopathy correction if needed, open fracture management begins with hemorrhage control using direct pressure or a tourniquet. If there is neurovascular compromise or significant pain, emergency department (ED) reduction should be attempted. Evaluation for vascular injury may include the ankle-brachial index or angiography. Grossly contaminated wounds should be irrigated, covered in sterile saline-soaked dressing, and splinted. Tetanus prophylaxis should be given to all patients. Timing to antibiotics is the most important determining factor in preventing infection. All type 1 or 2 open fractures (minimally to moderately contaminated) should receive a first-generation cephalosporin, irrigation, and debridement within 24 hours. All type 3 open fractures (severe soft tissue injury and/or arterial injury) should receive a first-generation cephalosporin, an aminoglycoside, and operative irrigation and debridement as soon as possible.

#### 3. Bafuma P. Boring Question: Does This Pediatric Patient Require a Hard Cast? *CanadiEM*. (March 23, 2015). http://canadiem.org/boring-question-does-this-pediatric-patient-require-a-hard-cast/

This blog post reviews the evidence supporting a removable splint for a non-displaced “buckle fracture” (aka torus fracture).

Take Home Points: The traditional recommendation for full immobilization with a cast or non-removable splint for buckle fractures appears unnecessary based on the best available evidence. A 2010 meta-analysis in the *Journal of Pediatric Orthopedics* revealed that 455 patients placed in removable splints did not suffer from re-fractures and had higher satisfaction scores.^14^ Additionally, multiple small studies showed better functional outcomes with either removable splints or simple ACE wraps. This review challenges the dogmatic teaching that full immobilization is necessary for optimal fracture healing and functional outcomes.

#### 4. Kivlehan S. PV Card: Adult Scaphoid Fracture. *Academic Life in Emergency Medicine.* (February 1, 2016). https://www.aliem.com/2016/pv-card-adult-scaphoid-fracture/

This post reviews the LR of different examination and radiograph findings to help diagnose this easily missed injury.^15^

Take Home Points: The scaphoid is the most commonly fractured carpal bone in adults. As radiographs have poor sensitivity, the physical examination may help identify this fracture. The two most powerful exam findings for suspected scaphoid fracture are the presence of resisted supination pain (positive LR 6.1, negative LR 0.09) and the clamp sign (positive LR 8.6, negative LR 0.4). Resisted supination pain is achieved by holding the patient’s injured hand and having him/her attempt forearm supination against examiner resistance. The clamp sign occurs when the patient identifies the painful area by placing thumb and forefinger of opposite hand on both sides of affected thumb. Commonly used examination findings like thumb compression pain (negative LR 0.24) and snuffbox tenderness (negative LR 0.15) do not achieve the desired negative LR threshold of <0.1. Scaphoid series radiographs at the time of injury are highly specific (100%) but only moderately sensitive (80%) for scaphoid fractures. CT and MRI both have strong positive LRs (15.4 and 22.0, respectively), but only MRI and bone scan have strong negative LR (0.09 and 0.11, respectively). These investigations are extremely important as missed scaphoid fractures can lead to avascular necrosis and nonunion.

#### 5. Helman A. Episode 52: Commonly Missed Uncommon Orthopedic Injuries. *EM Cases*. (October 1, 2014). http://emergencymedicinecases.com/episode-52-commonly-missed-uncommon-orthopedic-injuries/

This podcast and blog post reviews the most commonly missed orthopedic injuries and diagnostic recommendations.

Take Home Points: Four easily missed orthopedic injuries are Lisfranc fractures, perilunate injuries, distal radius-ulnar joint (DRUJ) injuries, and pelvic apophyseal avulsion fractures. Resulting from ankle external rotation with foot plantar flexion, Lisfranc fractures classically present with midfoot swelling, midfoot hematoma and/or ecchymosis on the plantar foot. Radiographs can be normal, though they may show subtle widening of >2 mm between the first, second or third metatarsal, or avulsion fractures of the medial cuneiform or second metatarsal. If a Lisfranc fracture is suspected, a 30-degree oblique radiograph, weight bearing radiograph, or CT should be performed. Treatment includes a posterior slab, non-weight bearing instructions, and outpatient orthopedic follow-up. An immediate orthopedic evaluation is indicated if the dislocation is >2 mm.

For perilunate injuries, the radiograph can show irregularities in the normally smooth Gilula lines, asymmetric intercarpal distances on the AP radiograph view, or a “slipped cup” sign on the lateral radiograph view. Obtaining radiographs of the uninjured wrist for comparison may be useful for equivocal cases. In DRUJ injuries, the AP wrist radiograph can show radial-ulna joint widening > 2 mm. Be sure to image the elbow as well, as radial head fractures may also be present. Treatment is closed reduction and a sugar-tong splint to prevent supination. Pelvic apophyseal fractures present similar to a muscle strain. They are important to diagnose as healing takes 6–8 weeks, and a non-weight bearing status may shorten healing times.

#### 6. Bafuma P. Clinical Question: How effective is intra-articular lidocaine for shoulder reduction? *CanadiEM* (March 22, 2016). http://canadiem.org/boring-question-effective-intra-articular-lidocaine-shoulder-reduction/

This post reviews the evidence for intra-articular (IA) lidocaine compared to procedural sedation to facilitate the reduction of shoulder dislocations.

Take Home Points: The post focuses on data from a 2011 Cochrane review^16^ and a 2014 *Journal of Clinical Anesthesia* meta-analysis.^17^ The Cochrane review found that IA lidocaine was as effective in shoulder reduction as intravenous analgesia with or without sedatives. There was no difference in pain reduction, and patients who received IA lidocaine had shorter ED stays. The meta-analysis similarly found equivalent shoulder reduction success rates as well as fewer complications (respiratory depression, vomiting, thrombophlebitis) in the IA lidocaine group. Neither study found an increase in joint infections with IA lidocaine. Overall, IA lidocaine is a reasonable pain management option for reducing shoulder dislocations.

#### 7. Mason R, St John A, Handy Knowledge: Subtle and High-Risk Hand Injuries. *EM Docs*. (September 15, 2015). http://www.emdocs.net/handy-knowledge-subtle-and-high-risk-hand-injuries/

This blog post reviews three high-risk hand injuries that should not be missed in the ED.

Take Home Points: Pyogenic flexor tenosynovitis should be suspected in penetrating, especially high velocity, injuries to the palmer surface of the hand. The physical exam (i.e. Kanavel’s signs) can be helpful in making this diagnosis, but all four signs are only present in about 50% of cases. Treatment includes emergent hand surgeon consultation and broad spectrum antibiotics that cover Staphylococcus, Streptococcus, and gram negative rods.

Hand compartment syndrome is commonly caused by fractures, penetrating injuries, and arterial injuries. Patients may present with pain on passive motion at the metacarpal-phalangeal (MCP) joint or with the hand in the intrinsic minus, or claw, position. Once the diagnosis is suspected, the compartment pressures should be measured in the suspected compartments. A fasciotomy is recommended when the compartment pressure is within 30 mmHg of the diastolic blood pressure.

Ulnar collateral ligament (UCL) disruption can occur with forceful radial movement of the thumb. Useful physical exam findings include a Stener lesion (tender swelling of the ulnar side at the base of the thumb representing the proximally displaced UCL), and increased ulnar laxity of the thumb MCP. To test for proper UCL tear, apply radial stress with the thumb in 30-degree flexion. For accessory UCL tear, apply radial stress with the thumb in 30-degree extension. If missed, this injury can result in permanent pincer strength weakness. Treatment includes immobilization with a thumb spica splint and follow up with a hand surgeon for possible surgical repair.

#### 8. Helman A. Episode 58: Tendons and Ligaments – Commonly Missed Uncommon Orthopedic Injuries Part 2. *EM Cases*. (January 1, 2015). http://emergencymedicinecases.com/episode-58-tendons-ligaments-missed-orthopedic-injuries/

The *EM Cases* team and two orthopedic surgeons present a podcast and written summary focusing on four high-risk orthopedic injuries of the tendons and ligaments.

Take Home Points: In ankle sprains, an injury to the tibia-fibula syndesmosis can occur. The Hopkins test, or pain near the talus on squeezing the tibia and fibula together at mid-calf, can help make the diagnosis. Ankle radiograph findings are subtle, but can include decreased tibio-fibular overlap, increased medial clear space, and increased tibio-fibular clear space. Treatment of this injury should include a non-weight bearing status, orthopedic follow up, and evaluation for other injuries such as fractures at the ankle, fifth metatarsal base, and proximal fibula.

Distal biceps tendon rupture occurs almost exclusively in young males and is commonly associated with heavy lifting. Exam findings include “Popeye” sign (a flexed, asymmetric biceps muscle), ecchymosis of the anterior aspect of the elbow, and the hook sign (no bicep tendon palpated by hooked finger on distal bicep). Surgical repair within two weeks is recommended for distal biceps tendon injury.

Every patient with knee pain or injury should perform a straight leg test. An inability to lift an extended leg is concerning for a quadriceps tendon or patella tendon rupture in the setting of normal knee radiographs. For these injuries, place the patient in a knee immobilizer with weight bearing as tolerated, and arrange urgent orthopedic follow up within a few days for surgical evaluation.

Gastrocnemius tears commonly occur from jumping or running up hill. Diagnosis and differentiation from Achilles’ tendon rupture is made using the calf raise test. Patients with gastrocnemius tears can perform a calf raise, but this motion reproduces the patient’s pain. In comparison, patients with Achilles’ tendon rupture cannot perform the calf raise test. Ultrasound may help the diagnosis, but radiographs are unrevealing. Treatment includes rest, ice, compression, and elevation, along with early weight-bearing exercises and physiotherapy.

#### 9. Tollins M, Johnson N. The Crashing Patient with Long Bone Fractures: A Case of Fat Embolism Syndrome. *EM Docs*. (January 21, 2016). http://www.emdocs.net/the-crashing-patient-with-long-bone-fractures-a-case-of-fat-embolism-syndrome/

This blog post reviews fat embolism syndrome (FES) including patient presentation, pathophysiology, diagnosis, and management.

Take Home Points: FES is a clinical diagnosis and should be suspected when patients with long bone fractures clinically deteriorate. The diagnosis can be made with one of three major and four of eight minor Gurd’s diagnostic criteria. Major criteria include the following: petechial rash, respiratory symptoms with radiographic changes, and central nervous system signs unrelated to trauma or other condition. Minor criteria include tachycardia, fever, retinal changes, renal abnormalities (oliguria, anuria, lipiduria), acute thrombocytopenia, acute hemoglobin decrease, elevated erythrocyte sedimentation rate, and fat globules in sputum. Classically, the brain MRI demonstrates a “star-field” pattern of punctate, hyperintense lesions. Treatment is supportive with steroids and statins lacking conclusive evidence. Though animal studies support heparin, it is not recommended in FES patients as concomitant polytrauma often increases the risk of complications from heparin. Patients frequently make significant improvements in neurologic status with supportive care.

#### 10. Brown J. Wrist and Distal Forearm Injuries: Pearls and Pitfalls. *EM Docs*. (November 7, 2015). http://www.emdocs.net/wrist-and-distal-forearm-injuries-pearls-pitfalls/

This post provides a comprehensive summary of the pearls, pitfalls, and management plans for common wrist and distal forearm injuries.

Take Home Points: Colles’ and Smith’s (reverse Colles’) fractures involve dorsally displaced and volarly displaced fractures of the radius, respectively. Both are reduced with longitudinal traction and placement of a sugar-tong or double sugar-tong splint. Distal radial fractures include Barton’s fractures, which extend through the joint space causing carpal bone dislocation, and Chauffeur’s (or Hutchinson’s) fractures, which are oblique fractures through the radial styloid process. Both are intra-articular and typically require open reduction and internal fixation.

Carpal bone fractures are challenging to diagnose. Because scaphoid fractures may not appear on initial radiographs and are at risk for avascular necrosis, patients with snuffbox tenderness should receive a thumb spica splint and orthopedic follow up. Triquetrum fractures appear as a small fracture fragment dorsal to the carpal bones on the lateral wrist radiograph. Treatment includes a short-arm splint with outpatient orthopedic follow up. For hamate fractures, an additional carpal tunnel view wrist radiograph may be helpful, but, if the clinical suspicion is high, a CT should be ordered. These fractures require an ulnar gutter splint and orthopedic follow up.

Ligamentous injuries of the wrist can result in scapholunate dissociation, perilunate dislocation, and lunate dislocation. In scapholunate dissociation, the AP view of the wrist radiograph may show the “Terry Thomas” sign (widening of the scapholunate space >2 mm). Perilunate and lunate dislocations can result in median nerve neuropathy, avascular necrosis of the carpal bones, and persistent wrist instability. These two injuries require immediate orthopedic consultation for open reduction and internal fixation.

#### 11. Fox S. Septic Arthritis. *Peds EM Morsels*. (August 28, 2015). http://pedemmorsels.com/septic-arthritis/

This blog post reviews the presentation, pathophysiology, workup, and treatment for septic arthritis in the pediatric population.

Take Home Points: Pediatric septic arthritis usually presents with fever (80%), pain with passive range of joint motion, and tends to involve the lower extremities (80%). *Staphylococcus aureus* is the most common organism, but others include *Streptococcus* species, *Escherichia coli*, *Salmonella*, and *Neisseria gonorrhea* in certain populations. The Kocher criteria – fever, non-weight bearing, ESR ≥40 mm/hr, and a serum WBC ≥12,000 cells/L - can help risk-stratify patients with a suspected septic hip joint, but lack robust validation. No single test can rule out septic arthritis.

#### 12. Fox S. Shoulder Dislocation. *Peds EM Morsels*. (July 31, 2015). http://pedemmorsels.com/shoulder-dislocation/

This blog post reviews the anatomy, presentation, evaluation, and management of pediatric shoulder dislocations.

Take Home Points: Although shoulder dislocations occur less frequently in children compared to adults, there are some key lessons. Physeal fractures are possible with dislocation (ossification centers close between age 5–7 years). If there is a low-energy mechanism and dislocation is clinically apparent, imaging may not be necessary. It is important to maintain a low threshold to image skeletally immature patients <14 years old. Intranasal analgesia or ultrasound-guided intra-articular injection may aid in pain control. All cases should follow up with orthopedics as it is unclear whether physical therapy alone versus surgical stabilization is superior.

#### 13. Shenvi C. Hip Fractures in Older Adults: An Important Source of Morbidity. *Academic Life in Emergency Medicine*. (September 15, 2015) https://www.aliem.com/2015/hip-fractures-in-older-adults/

This blog post focuses on risk factors, diagnosis, and management of acute hip fractures in the elderly population.

Take Home Points: For elderly patients, hip fractures increase their one-year mortality twofold, and half of surviving patients will not return to a pre-injury level of function. Although hip radiographs usually identify the fracture, occult hip fractures may not show on radiograph. If the patient cannot bear weight on the injured extremity or there is a high clinical suspicion for a fracture, obtain an MRI or CT. In addition to an orthopedic consult, the emergency physician must assess the patient for concurrent injuries as well as evaluate for dangerous etiologies, such as syncope, that contributed to the fall. Opioids can be given for analgesia, but femoral or fascia iliaca nerve blocks should be considered.

#### 14. Faust J. Westafer L. Episode 40 - Femoral Nerve Blocks & Compartment Syndrome. *FOAMcast*. (December 23, 2015). http://foamcast.org/2015/12/23/episode-40-femoral-nerve-blocks-compartment-syndrome/

This podcast and blog post reviews Dr. Ken Milne’s podcast on regional anesthesia for hip fractures based on the 2016 *Canadian Journal of Emergency Medicine* systematic review publication by Ritcey et al.^13^

Take Home Points: Elderly patients with hip fractures require analgesia, but parenteral dosing is difficult as under-dosing must be balanced against the potential for medication adverse effects like hypotension, allergic reactions, over-sedation, and delirium. Regional nerve blocks have been demonstrated to be effective. Unfortunately, barriers to nerve block implementation in the ED include knowledge translation, skill acquisition, and lack of time on a busy shift. Furthermore, orthopedic consultants may be concerned that nerve blocks can mask compartment syndrome, though this concern is largely unfounded. To review, compartment syndrome classically presents with the 5 P’s - pain, paresthesia, pallor, pulselessness, and poikilothermia – though these are unreliable. Compartment pressures >30 mmHg or a delta pressure (diastolic pressure minus the compartment pressure) <20–30 mmHg are concerning for compartment syndrome. Treatments include fasciotomy and surgery consult.

## CONCLUSION

The ALiEM Blog and Podcast Watch series serves to identify educational quality blogs and podcasts for EM clinicians through its expert panel using an objective scoring instrument. These social media resources are currently curated in the ALiEM AIR and AIR-Pro Series, originally created to address EM residency needs. These resources are herein shared and summarized to help clinicians filter the rapidly published multitude of blog posts and podcasts. Limitations include the search only includes content produced within the previous 12 months from the top 50 Social Media Index sites. While these lists are by no means a comprehensive analysis of the entire Internet for these topics, this series provides a post-publication accreditation and curation of recent, online content to identify and recommend high quality, educational social media content for the EM clinician.

## Figures and Tables

**Table:** Approved Instructional Resources (AIR) scoring instrument for blog and podcast content with the maximum score being 35 points.

Tier 1: BEEM rater scale	Score	Tier 2: content accuracy	Score	Tier 3: educational utility	Score	Tier 4: evidence based medicine	Score	Tier 5: referenced	Score
Assuming that the results of this article are valid, how much does this article impact on EM clinical practice?		Do you have any concerns about the accuracy of the data presented or conclusions of this article?		Are there useful educational pearls in this article for senior residents?		Does this article reflect evidence based medicine (EBM)?		Are the authors and literature clearly cited?	
Useless information	1	Yes, many concerns from many inaccuracies	1	Not required knowledge for a competent EP	1	Not EBM based, only expert opinion	1	No	1
Not really interesting, not really new, changes nothing	2		2		2		2		2
Interesting and new, but doesn’t change practice	3	Yes, a major concern about few inaccuracies	3	Yes, but there are only a few (1–2) educational pearls that will make the EP a better practitioner to know or multiple (>=3) educational pearls that are interesting or potentially useful, but rarely required or helpful for the daily practice of an EP.	3	Minimally EBM based	3		3
Interesting and new, has the potential to change practice	4		4		4		4	Yes, authors and general references are listed (but no in-line references)	4
New and important: this would probably change practice for some EPs	5	Minimal concerns over minor inaccuracies	5	Yes, there are several (>=3) educational pearls that will make the EP a better practitioner to know, or a few (1–2) every competent EP must know in their practice	5	Mostly EBM based	5		5
New and important: this would change practice for most EPs	6		6		6		6		6
This is a “must know” for EPs	7	No concerns over inaccuracies	7	Yes, there are multiple educational pearls that every competent EP must know in their practice	7	Yes exclusively EBM based	7	Yes, authors and in-line references are provided	7

*BEEM,* best evidence in emergency medicine; *EM,* emergency medicine; *EP,* emergency physician.
